# A case of gallbladder abscess caused by torsion that completely disappeared following drainage

**DOI:** 10.1016/j.radcr.2024.06.037

**Published:** 2024-07-06

**Authors:** Rika Yoshida, Shota Tanaka, Tomonori Nakamura, Anna Murata, Shota Kato, Hideyuki Nagai, Takeshi Yoshizako, Kazuhiro Kitajima, Yasushi Kaji

**Affiliations:** a Shimane University, Faculty of Medicine, Department of Radiology, 89-1, Enyacho, Izumoshi, Shimane, 693-8501, Japan; b Matsue Seikyo General Hospital, Department of Radiology, 8-8-8, Nishitsuda, Matsueshi, Shimane, 690-8522, Japan; c Hyogo Medical University, Department of Radiology, 1-1, Mukogawacho, Nishinomiyashi, Hyogo, 663-8501, Japan

**Keywords:** Acute abdomen, Computed tomography (CT), Gallbladder torsion, Gallbladder volvulus

## Abstract

A woman in her 90s presented with loss of appetite, lower back pain, abdominal pain, and fever. Physical examination and subsequent blood tests indicated an inflammatory process, and computed tomography (CT) scans revealed gallbladder torsion with necrosis and abscess formation. The case involved successful management of this rare condition through percutaneous drainage without the need for surgical intervention, avoiding complications during follow-up. Remarkably, post-treatment CT showed complete resolution of the gallbladder abscess and the gallbladder itself was no longer visible. This case highlights the effectiveness of minimally invasive treatment for gallbladder torsion in elderly patients and underscores the potential for non-surgical intervention in managing complex abdominal conditions.

## Introduction

Gallbladder torsion, or twisted gallbladder, is a rare acute abdominal condition primarily in individuals with a floating gallbladder [[1], [2], [3], [4]] that typically presents with sudden right upper quadrant pain, nausea, and vomiting and more commonly occurs in thin elderly women [[1], [2], [3]]. A twisted gallbladder can sometimes be palpated as a mass in the right upper quadrant and may occur due to acquired factors, such as kyphosis, scoliosis, thinness, or abdominal trauma. This report presents a case of intraabdominal abscess, caused by a rare acute condition of gallbladder necrosis due to torsion, improved with drainage. Remarkably, the gallbladder, including the abscess, was completely resolved without surgical intervention. This case report is presented with a literature review.

## Case report

A woman in her 90s experienced a loss of appetite for a month. A few days before her presentation, she developed back pain, abdominal pain, and fever and finally visited our hospital. Physical examination revealed tenderness from the right upper quadrant to the lower abdomen with increased pain during movements and right hip flexion.

Leukocytosis (13,700/µL) and elevated inflammatory markers (C-reactive protein, 13.6 mg/dl) were found in laboratory findings. Hepatobiliary enzymes were within normal limits.

The patient received oxygen therapy due to hypoxemia caused by hypertension and heart failure.

**Fig. 1:**
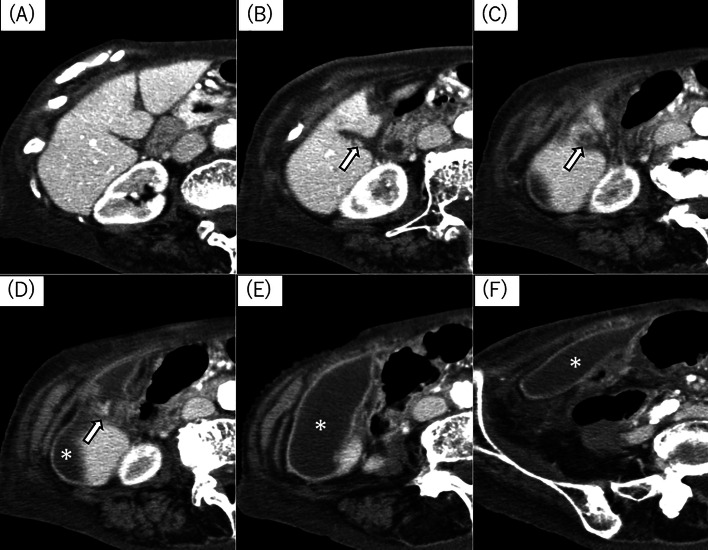
Abdominal computed tomography (CT) (axial image, A to F is arranged from the head to tail, respectively). Contrast-enhanced CT scan revealed an encapsulated fluid collection of >10 cm with a wall structure extending from the right upper quadrant to the right lower abdomen. The normal gallbladder was not visualized. A whirl sign observed in the CT indicates the torsion at the gallbladder neck (Fig. 1, arrow), with the body and base of the gallbladder being completely unidentifiable and appearing continuous with the encapsulated fluid collection (Fig. 1,*).

A noncontrast-enhanced CT scan was performed to determine a potential source of abdominal infection and revealed an encapsulated fluid collection of >10 cm with a wall structure extending from the right upper quadrant to the right lower abdomen. However, the normal gallbladder was not visualized. Contrast-enhanced CT revealed torsion at the gallbladder neck, with the body and base being completely unidentifiable and appearing continuous with the encapsulated fluid collection (Fig. 1).

**Fig. 2:**
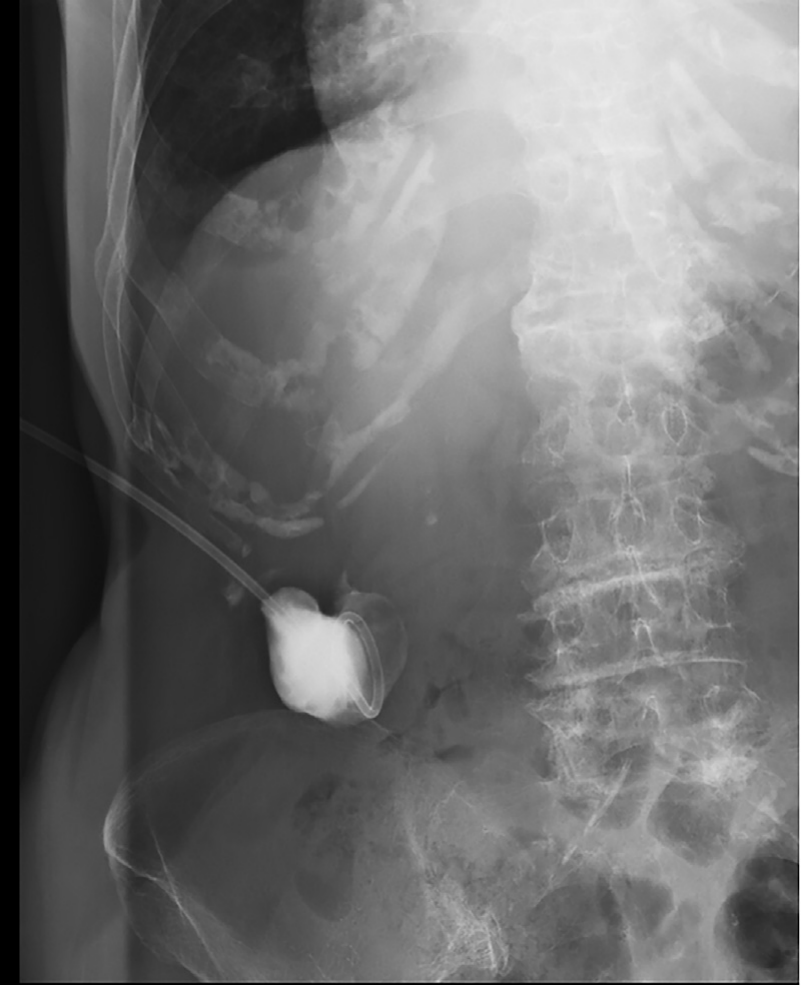
Contrast image confirming the drainage abscess cavity. An abdominal X-ray shows the drainage abscess cavity (Fig. 2).

**Fig. 3:**
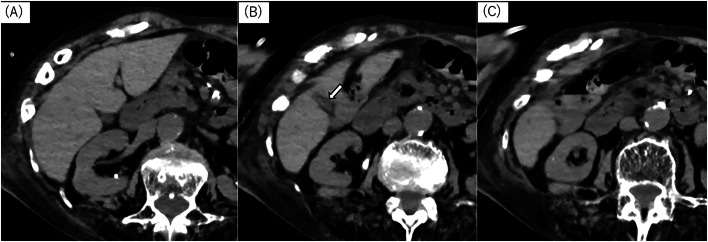
Follow-up noncontrast CT, axial image. Follow-up noncontrast CT confirmed that not only the abscess but also the gallbladder disappeared without biliary dilatation or bile leakage (Fig. 3, arrow).

The patient was diagnosed with gallbladder torsion and necrotic abscess. Considering the risks associated with general anesthesia due to reduced cardiac function and severe hiatal hernia with compromised pulmonary function, ultrasound-guided percutaneous drainage (8.5-Fr Dawson-Mueller drainage catheter®; Cook Canada Inc., Stouffville, Ontario, Canada) was performed. The causative organism was identified as *Klebsiella pneumoniae* (Fig. 2). The patient responded well to intravenous antibiotics and drainage, which reduced and resolved the abscess. No complications, such as biliary fistula or bile duct dilatation, were noted, and no symptom recurrence was reported over 3 years. The noncontrast CT scan, performed 3 years later during the investigation of a fever, confirmed the disappearance of both the abscess and the gallbladder (Fig. 3). This CT also established that the cause of the fever at that time was aspiration pneumonia.

## Discussion

Gallbladder torsion, or gallbladder volvulus, typically presents with acute right upper quadrant pain, nausea, and vomiting, and commonly occurs in thin elderly women [[1], [2], [3], [4], [5], [6], [7], [8]]. The twisted gallbladder can sometimes be palpated as a mass in the right upper quadrant and can occur due to acquired factors, such as kyphosis, scoliosis, being underweight, or abdominal trauma. Gallbladder torsion is a rare acute abdominal condition due to a floating gallbladder. The gallbladder is normally anchored to the liver by the connective tissues in the liver's fibrous capsule. A floating gallbladder (mobile gallbladder) is defined as short fixation, which can lead to torsion. Gross classified floating gallbladders into two types: type I, where the gallbladder is attached only by the gallbladder mesentery away from the liver, and type II, where only the cystic duct is slightly fixed [6].

Imaging findings in gallbladder torsion reveal that the gallbladder is detached and displaced from the gallbladder bed. Tumor-like shadows, including the neck or cystic duct of the gallbladder, can also be seen, with a swirling pattern (whirl sign) indicating torsion [[7], [8], [9]]. Gallbladder opacification is poor in complete torsion, whereas the arterial system remains patent and is accompanied by congestion in incomplete torsion, although the venous system is obstructed. Gallbladder wall thickening and enlargement can also be observed. High-density gallbladder mucosal surface was detected in noncontrast-enhanced CT, indicating microhemorrhages due to mucosal hemorrhagic necrosis. These imaging findings are considered to be in the acute phase. When gallbladder torsion is diagnosed, cholecystectomy is generally performed [[1], [2], [3], [4], [5],[7], [8], [9], [10]].

In this case, the patient was an elderly woman, the typical age group that predominantly suffers from this condition. No acute abdominal pain was reported, but she gradually developed discomfort in the right upper quadrant for 1 month. Her condition likely started with an incomplete torsion that slowly progressed to gallbladder necrosis, rupture, and eventual detachment, resulting in abscess formation that extended from the right upper quadrant to the lower abdomen. Although the whirl sign at the gallbladder neck was confirmed, indicating gallbladder torsion necrosis, surgery was not performed due to the patient's overall condition. The actual gallbladder status thus remains unknown, a limitation of this case. The cystic duct and gallbladder neck were twisted and eventually completely occluded, resulting in the disappearance of the gallbladder itself after the drainage without any bile leakage. This is a case of an intraperitoneal abscess caused by gallbladder torsion and necrosis, which improved with drainage and was resolved without surgical intervention, including the gallbladder disappearance. A literature review was also performed. This finding illustrates that drainage might be a viable treatment option for elderly patients where surgery is challenging.

## Conclusion

Drainage improved the intraperitoneal abscess caused by gallbladder torsion and necrosis without the need for any surgical intervention, leaving no trace of the gallbladder.

## Patient consent

Informed consent was obtained for the publication of this case report.
